# Supplementary material for: MEX3A regulates Lgr5 + stem cell maintenance in the developing intestinal epithelium
Source: EMBO Rep. 2020 Feb 13;21(4):e48938. doi: 10.15252/embr.201948938 (PMC7132344; doi:10.15252/embr.201948938)
Supplement: Supplementary file 1 — Appendix [file EMBR-21-e48938-s001.pdf]

Table of contents

Appendix Figure S1: Macroscopic assessment of major organs in the *Mex3a* mutant mice ………………………………………………………………………….. 2

Appendix Figure S2: *Mex3a* deletion leads to histological alterations in the proximal small intestine ………………………………………………………………… 3

Appendix Figure S3: Reduction in the number of proliferating cells upon *Mex3a* deletion impacts crypt size ……………………………………………………………….. 4

Appendix Figure S4: *Mex3a* KO cells exhibit a delayed transition from spheroid to budding organoid stage ……………..……………………………………………………. 5

Appendix Figure S5. MEX3A regulates PPARγ expression in cancer cell lines … 6

Appendix Figure S6. Caco-2 MEX3A-overexpressing cells present low metabolic output ……………………………………………………………………………………. 8

Appendix Table S1. List of primers used in this study ……………………………… 10

Appendix Table S2. List of antibodies used in this study ……………………………12

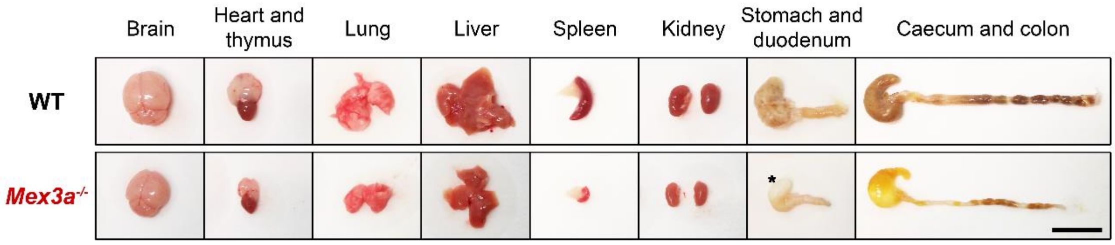

Appendix Figure S1: Macroscopic assessment of major organs in the *Mex3a* mutant mice.

Representative images of the different organs of a *Mex3a* null mouse and a littermate control are shown for size comparison. In accordance with body mass, all organs of the *Mex3a* null animal are smaller compared to the control. Of note, milk (*) is still visible in the stomach of the mutant mouse at this stage (P21). Scale bar, 1 cm.

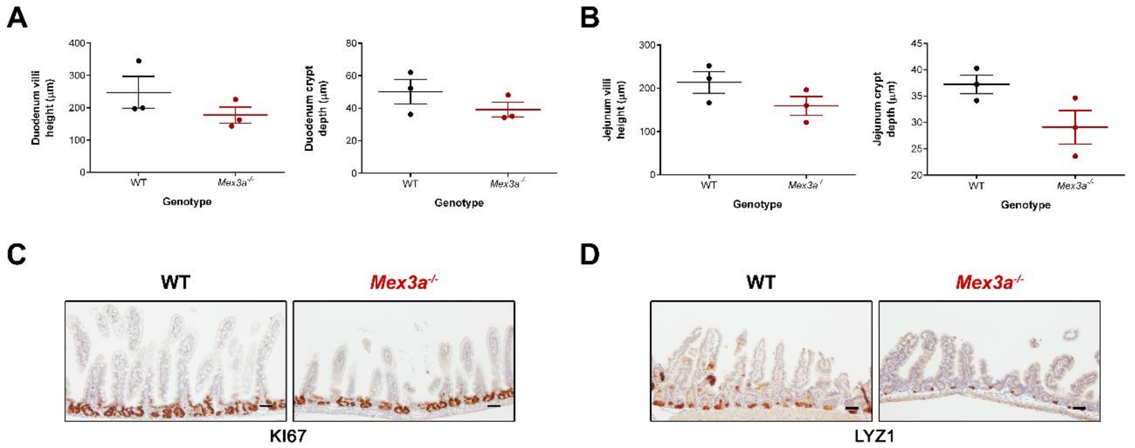

Appendix Figure S2: *Mex3a* deletion leads to histological alterations in the proximal small intestine.

A and B. Average villi height and crypt depth in (A) duodenum and (B) jejunum of WT and *Mex3a* KO animals. Data are presented as mean ± standard error (n = 3 for each genotype, > 10 villi and > 20 crypts counted per animal).

C and D. Representative immunohistochemistry staining for (C) the proliferation marker KI67 and (D) the Paneth cell marker LYZ1 in the proximal jejunum of WT and *Mex3a* KO mice. Scale bars, 50 μm.

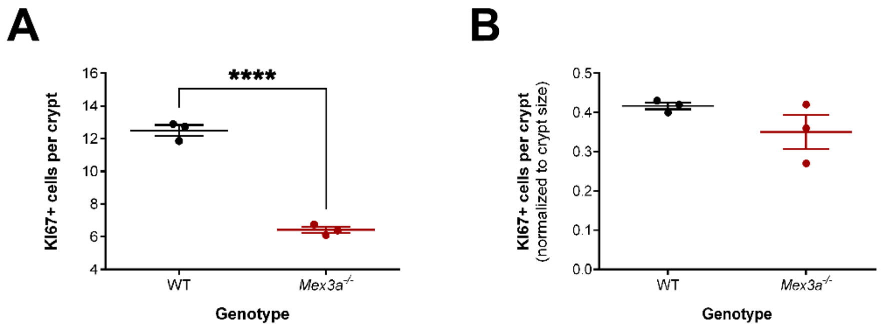

Appendix Figure S3: Reduction in the number of proliferating cells upon *Mex3a* deletion impacts crypt size

A. Average KI67+ cells per crypt in WT versus *Mex3a* KO animals.

B. Number of KI67+ cells normalized to crypt size in WT versus *Mex3a* KO animals.

Data information: Data are presented as mean ± standard error (n = 3 for each genotype, > 20 crypts counted per animal). ****$P < 0.0001$, Student`s *t* test.

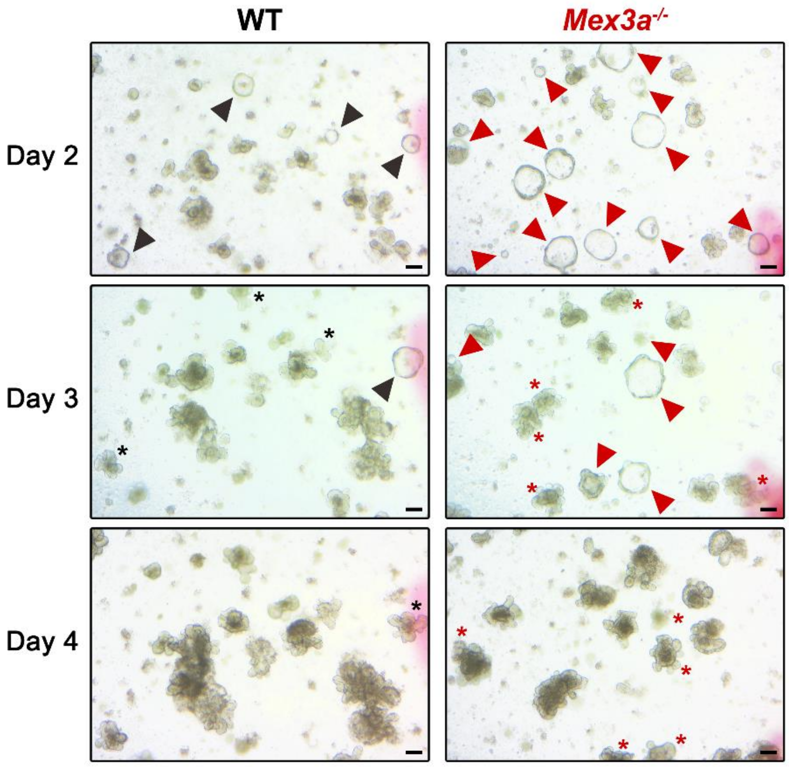

Appendix Figure S4: *Mex3a* KO cells exhibit a delayed transition from spheroid to budding organoid stage

Representative phase contrast microscopy images of intestinal organoids generated from *Mex3a* KO and WT cells from day 2 to day 4 of culture. A cohort of individual *Mex3a* KO spheroids (red arrowheads) shows delayed conversion into budding organoids (red asterisks) when compared to WT (black arrowheads and black asterisks), which are already present in a reduced number at the starting point. Scale bars, 100μm

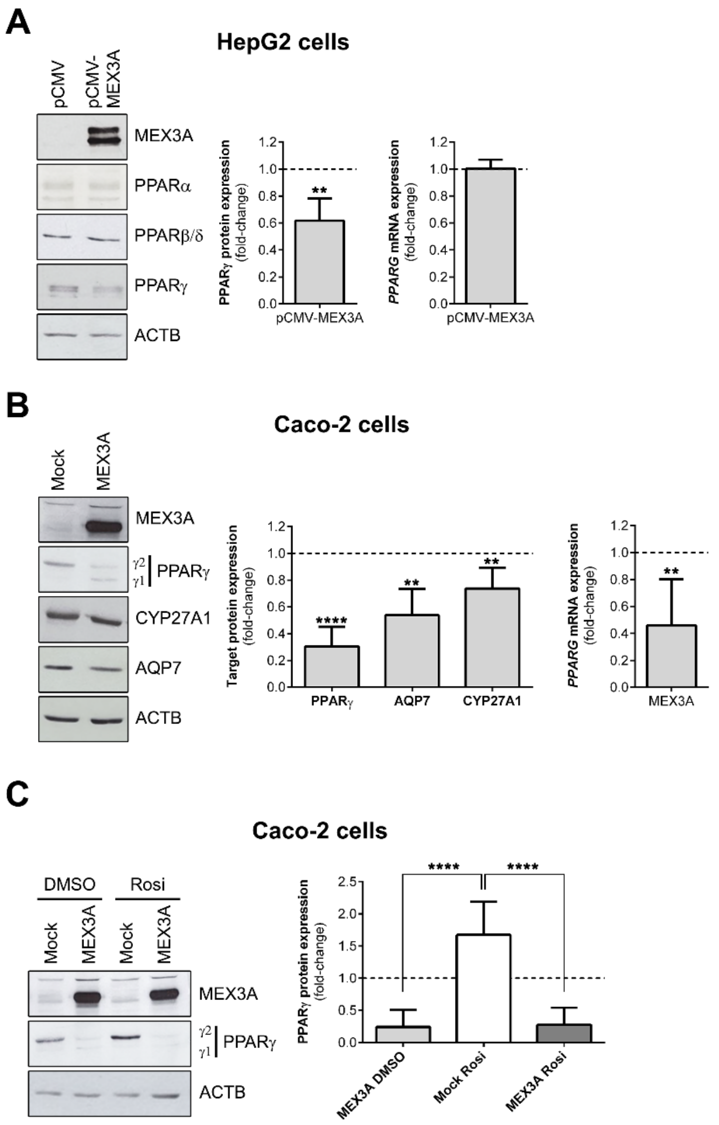

Appendix Figure S5. MEX3A regulates PPARγ expression in cancer cell lines

A. Representative western blots of MEX3A transient overexpression experiments in the HepG2 cell line. Data is presented as mean fold-change with 95% confidence interval (n = 3) for PPARγ protein *PPARG* mRNA expression levels in pCMV-MEX3A transfected cells relative to empty-vector (pCMV) transfected cells (dashed lines). ***P* = 0.0099, Student`s *t* test.

B. Representative western blots of MEX3A stable overexpression in Caco-2 cells. The two main PPARγ isoforms (γ1 and γ2) are clearly distinguishable. Data is presented as mean fold-change with 95% confidence interval (n = 6) for PPARγ2, AQP7 and CYP27A1 protein levels, as well as for *PPARG* mRNA levels in MEX3A-overexpressing cells relative to mock cells (dashed lines). *****P* < 0.0001, ***P* < 0.01, Student`s *t* test.

C. Representative western blots of Caco-2 cells treated with 20 μM Rosiglitazone or vehicle-treated with DMSO. Data is presented as mean fold-change with 95% confidence interval (n = 4) for PPARγ2 protein expression relative to vehicle-treated mock cells (dashed line). ****$P < 0.0001$, two-way ANOVA test.

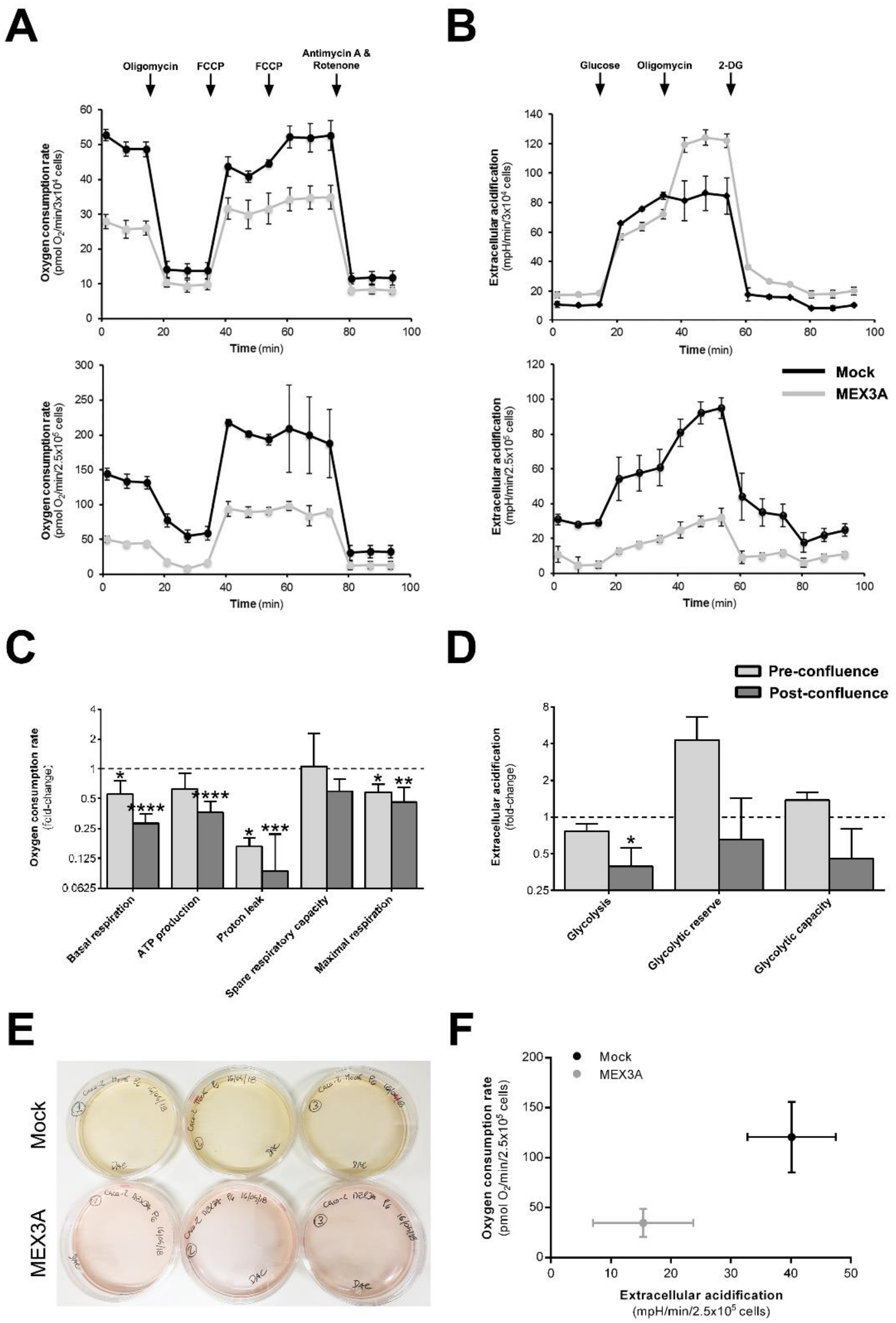

Appendix Figure S6. Caco-2 MEX3A-overexpressing cells present low metabolic output

A and B. Representative measurements of oxygen consumption (A) and extracellular acidification rate (B) in Caco-2 cells during pre-confluent (upper panels) or post-confluent (lower panels) cell culture conditions using the Seahorse Bioscience XF96 Extracellular Flux Analyzer.

C and D. The relative levels of key parameters of mitochondrial (C) and glycolytic (D) functions were calculated based on the measurements obtained upon addition of the

compounds indicated above (FCCP: Carbonyl cyanide-4 [trifluoromethoxy] phenylhydrazone; 2-DG: 2-deoxy-glucose). Data is presented as mean fold-change with range (n = 3 for pre-confluence and n = 5 for post-confluence) of MEX3A-overexpressing cells relative to mock cells (dashed line). *$P < 0.05$, **$P < 0.01$, ***$P < 0.001$, ***$P < 0.001$, Student`s *t* test.

E. Difference in culture medium pH between mock and MEX3A-overexpressing cells during post-confluent culture conditions.

F. Energy map depicting basal oxygen consumption and glycolysis levels of mock and MEX3A-overexpressing cells in post-confluent culture conditions.

Appendix Table S1. List of primers used in this study

Expression

Gene	Sense	Antisense
Apoa1	ACAGCTGAACCTGAATCTCCT	ATCCCAGAAGTCCCGAGTCA
Angptl4	ATCTTCAGAGCCAGATAGACC	AGCTGGGTCATCTTGGGAAG
Aqp7	CCTGGATGAGGCATTCGTGAC	CCCTTGAAGTGCTGGACTGT
Axin2	TGGCCAGTCAGCAGAGGGACA	TTTGGCTCTTTGTGATCTTCTGG
Ccnd1	CTGCTGCAAATGGAACTGCTTC	GCTTGTTCTCATCCGCCTCT
Cd36	GGCCAAGCTATTGCGACATG	ACAGCGTAGATAGACCTGCA
Cidec	AGGTCGCTGTCCAGGCATGT	ATCTGCGGTGCTAACACGA
Cyp27a1	TGGACACGACATCCAACAC	ATGTGGGCAAAGTCCTTGTGC
Egf	GGCATGAACTATGAAGATGAC	CCCTGGCAAACCCTTTCAGA
Fzd2	TCCTCACATGGTCGGTGTTG	GTGTAGCAGCCGGACAGAAAG
Hopx	AGACGCAGAAATGGTTTAAGC	TCCAAGAGCAAGCTCAAGGG
Kcne3	CTGAACTGTTTGATCACATTCCA	TACCAGGTCTCAGTCCCGTT
Lgr5	AGCGTCTTCACCTCCTACCTG	CTTGGGAATGTGTGTCAAAGC
Lrig1	CAAGCTGACTCTGTTTGGAAAC	ACTGGACAGACCTGATTGC
Lyz1	ACAATCGTTGTGAGTTGGCCAG	TAAACACACCCAGTCAGCCAG
Nog	CCATCCAAGTCTGTGCACCT	GAGTTCTAGCAGGAACACTTAC
Olfm4	TTCTAAGGTGAAGGAGTATGTC	ATGCTGTCCTTCTCCATGACC
Pdk4	AGTCCAAGATGCCTTTGAGTG	TTTCCATTGACTTGTGTGAGG
Pcna	GGGCTGAAGATAATGCAGACA	TGTACTCCTGTTCTGGGATTC
Pparg	CGGACAAATCACCATTTGTCATC	GATGGCCACCTCTTTGCTCT
Prelp	AGCATAGAGAAAATCAACGGGA	TGGGATGGGAGGCTTCAGAA
Rspo1	GCCACAACTTCTGCACCAAG	ATTTCACATTGTGCAGGACTG
Wnt2b	AGGCTGCAGGTTCCCTAGGTA	GGTGACCCGAGTTGTGTCAT
Wnt3	CCTGTCTTGGACAAAGCCAC	ATGTGAGTCACAGCCGCAGATG
18S	CGCCGCTAGAGGTGAAATTC	CATTCTTGGCAAATGCTTTCG
PPARA	GCAATCCATCGGCGAGGATA	CTGGTGAAAGCGTGTCCGTG
PPARD	AAAAGTTTTGGCAGGAGCGGG	ACTGTACAACACTGTCCCGGC
PPARG	CAGACAAATCACCATTCGTTATC	GATGGCCACCTCTTTGCTCT

Genotyping

Gene	Sense	Antisense
Mex3a_WT	TGCAGGGTTTCTCTAAACTGG	ACCAGGGACATGGAGCTTAG
**Mex3a_LacZ**	ATCCTCTGCATGGTCAGGTC	CGTTACGCGTTCGCTCATC
Lgr5_WT	CTGCTCTCTGCTCCCAGTCT	ATACCCCATCCCTTTTGAGC
Lgr5_KI	CTGCTCTCTGCTCCCAGTCT	GAACTTCAGGGTCAGCTTGC
Meox2_WT	GGGACCACCTTCTTTTGGCTTC	AAGATGTGGAGAGTTCGGGGTAG
Meox2_KI	GGGACCACCTTCTTTTGGCTTC	CCAGATCCTCCTCAGAAATCAGC

Appendix Table S2. List of antibodies used in this study

Antigen	Species	Dilution (assay)[a]	Reference	RRID code	Source
ACTB	Goat	1:2000 (WB)	sc-1616	AB_630836	Santa Cruz Biotech.
AQP7	Mouse IgG_{2a}	1:500 (WB)	sc-376407	AB_11149931	Santa Cruz Biotech.
BrdU	Mouse IgG_1 (Clone Bu20a)	1:75 (IF)	M0744	AB_10013660	Dako
CDX2	Mouse IgG_1 (Clone CDX2-88)	1:50 (IHC)	MU392A-UC	AB_2650531	Biogenex
CYP27A1	Mouse IgG_{2a}	1:500 (WB)	sc-390974	-	Santa Cruz Biotech.
DCKL1	Rabbit	1:2000 (IHC)	ab31704	AB_873537	Abcam
GFP	Mouse IgG_{2a}	1:250 (IHC)	sc-9996	AB_627695	Santa Cruz Biotech.
KI67	Rabbit (Clone SP6)	1:1000 (IF and IHC)	ab16667	AB_302459	Abcam
LYZ1	Rabbit	1:1500 (IHC)	A0099	AB_2341230	Dako
MEX3A	Rabbit	1:2000 (WB)	PRS4869	AB_1853839	Sigma
PPARα	Mouse IgG_{2b} (Clone 3B6/PPAR)	1:500 (WB)	MA1-822	AB_2165745	Thermo Fisher Scientifc
PPARβ	Rabbit	1:4000 (WB)	PA1-823A	AB_2165895	Thermo Fisher Scientifc
PPARγ	Rabbit (Clone C26H12)	1:300 (IHC); 1:1000 (WB)	#2435	AB_2166051	Cell Signalling Technology
SYP	Rabbit (SP11)	1:100 (IHC)	RM-9111-S0	AB_149939	Thermo Fisher Scientifc
VIL1	Mouse IgG_1 (Clone 1D2C3)	1:750 (IHC)	sc-58897	AB_2304475	Santa Cruz Biotech.
Anti-Rabbit (Biotinylated)	Swine	1:100 (IHC)	E0353	AB_2737292	Dako
Anti-Mouse (Biotinylated)	Rabbit	1:100 (IHC)	E0354	AB_2687571	Dako

Anti-Mouse (Alexa Fluor 594 conjugated)	Goat	1:100 (IF)	A-11032	AB_141672	Thermo Fisher Scientifc
Anti-Rabbit (Alexa Fluor 488 conjugated)	Goat	1:100 (IF)	A-11034	AB_2576217	Thermo Fisher Scientifc
Anti-Goat (HRP conjugated)	Donkey	1:2000 (WB)	sc-2020	AB_631728	Santa Cruz Biotech.
Anti-Mouse (HRP conjugated)	Goat	1:2000 (WB)	sc-2005	AB_631736	Santa Cruz Biotech.
Anti-Rabbit (HRP conjugated)	Goat	1:2000 (WB)	sc-2004	AB_631746	Santa Cruz Biotech.

a) IF - Immunofluorescence; IHC - Immunohistochemistry; WB - Western blot
