# Supplementary material for: MEX3A regulates Lgr5 + stem cell maintenance in the developing intestinal epithelium
Source: EMBO Rep. 2020 Feb 13;21(4):e48938. doi: 10.15252/embr.201948938 (PMC7132344; doi:10.15252/embr.201948938)
Supplement: Supplementary file 2 — Expanded View Figures PDF [file EMBR-21-e48938-s002.pdf]

Expanded View Figures

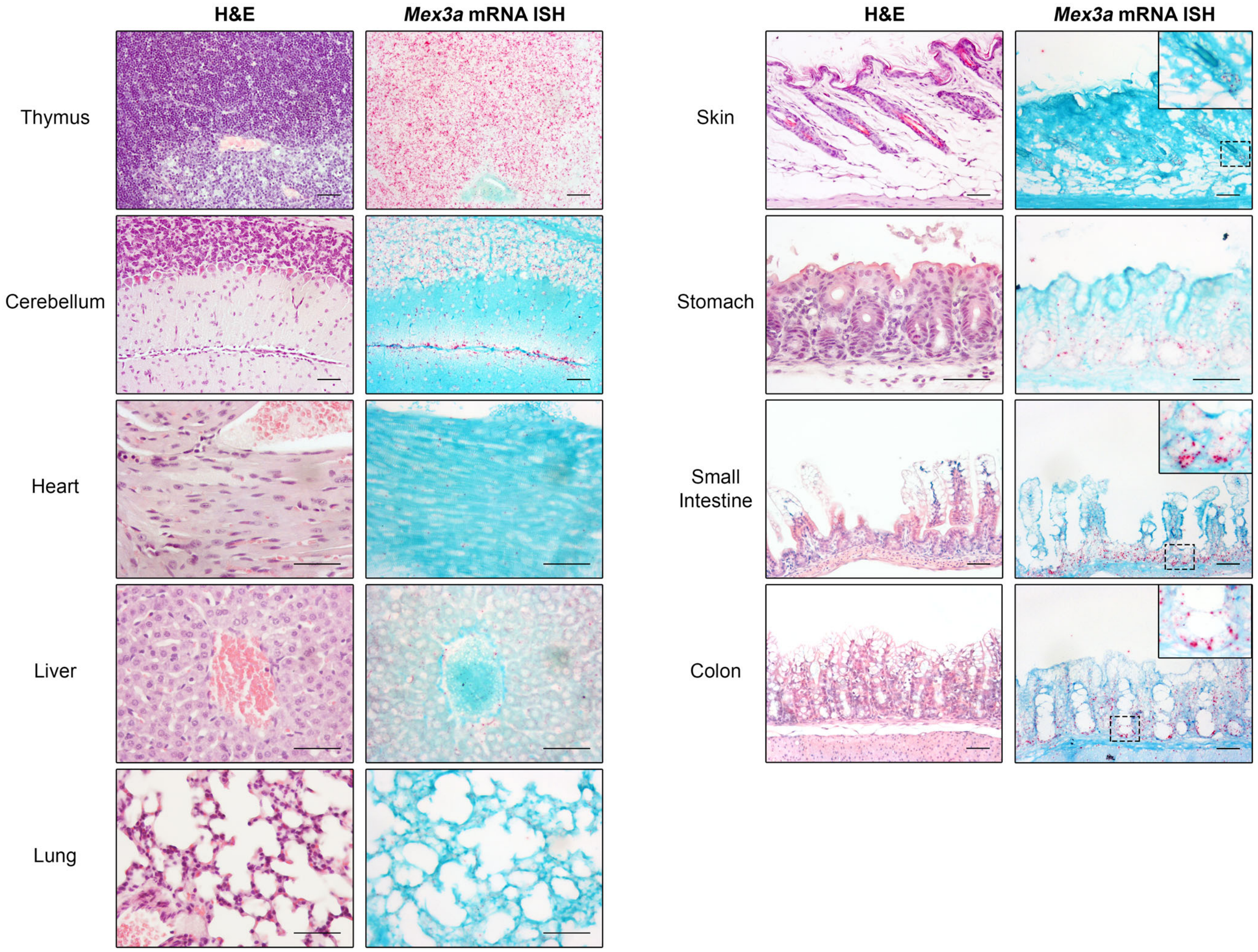

Figure EV1. Characterization of *Mex3a* expression pattern in murine tissues.

H&E staining and *Mex3a* mRNA ISH in serial sections of different mouse organs at postnatal day 17. Each punctuate red dot in the ISH panels represents a hybridization event with a single *Mex3a* mRNA molecule. Inserts depict high magnification of the boxed areas. The diffuse signals observed in the liver are the result of non-specific staining. Scale bars, 50 μm.

 © 2020 The Authors

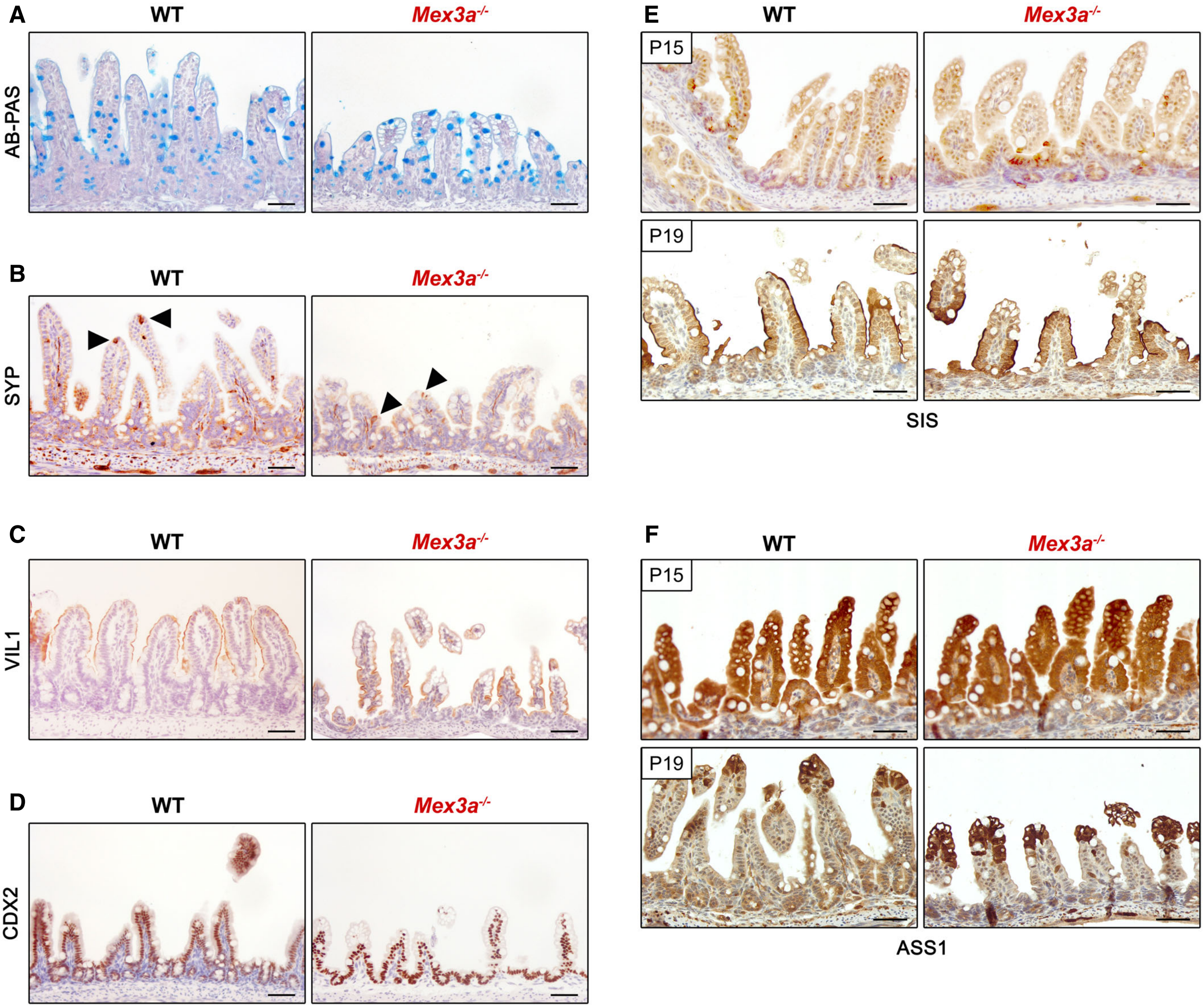

Figure EV2. *Mex3a* mutant mice do not exhibit a premature maturation of the intestinal epithelium.

A–C The main differentiated cell types are present in the *Mex3a* mutant mice intestinal epithelium, namely goblet cells as assessed by (A) alcian blue–periodic acid–Schiff (AB-PAS) reaction, (B) enteroendocrine cells as assessed by Synaptophysin (SYP, black arrowheads) expression and (C) enterocytes as assessed by Villin (VIL1) staining.

D Immunohistochemical staining for the intestinal transcription factor CDX2.

E Immunohistochemistry shows that sucrase-isomaltase (SIS) expression at the brush border is only observed at the suckling-to-weaning transition in the *Mex3a* mutant mice, as well as in the control.

F Expression of the suckling period-specific enzyme argininosuccinate synthetase 1 (ASS1) is also observed in the correct time-point.

Data information: All scale bars, 50 μm.

© 2020 The Authors

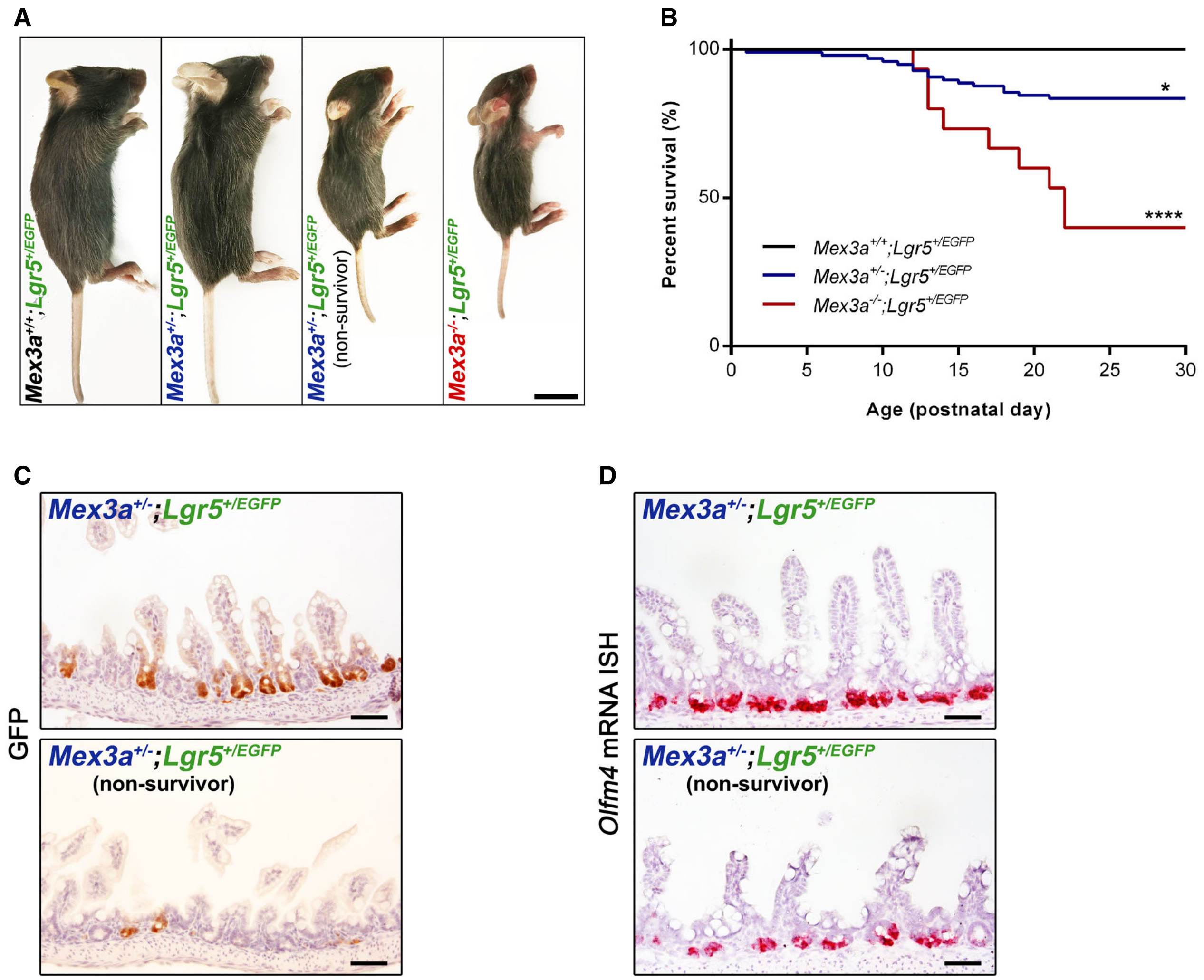

Figure EV3. Characterization of *Mex3a*$^{+/-}$;*Lgr5*$^{+/EGFP}$ double heterozygous mice.

A Representative images of the size of *Mex3a*$^{+/-}$;*Lgr5*$^{+/EGFP}$ (non-survivor), *Mex3a*$^{-/-}$; *Lgr5*$^{+/EGFP}$ and control littermates at P19. Scale bar, 1 cm.

B Kaplan–Meier survival curves for the indicated genotypes (*Mex3a*$^{+/+}$;*Lgr5*$^{+/EGFP}$, n = 27; *Mex3a*$^{+/-}$;*Lgr5*$^{+/EGF}$, n = 97; *Mex3a*$^{-/-}$;*Lgr5*$^{+/EGFP}$, n = 15). *P = 0.03, ****P < 0.0001, log-rank (Mantel–Cox) test.

C Immunohistochemical staining for GFP in ileal sections of *Mex3a*$^{+/-}$;*Lgr5*$^{+/EGFP}$ compound mice (non-survivor versus survivor littermate) at P17.

D *Olfm4* mRNA ISH in ileal sections of *Mex3a*$^{+/-}$;*Lgr5*$^{+/EGFP}$ compound mice (non-survivor versus survivor littermate) at P22.

Data information: Scale bar (A), 1 cm; all other scale bars, 50 µm.

© 2020 The Authors

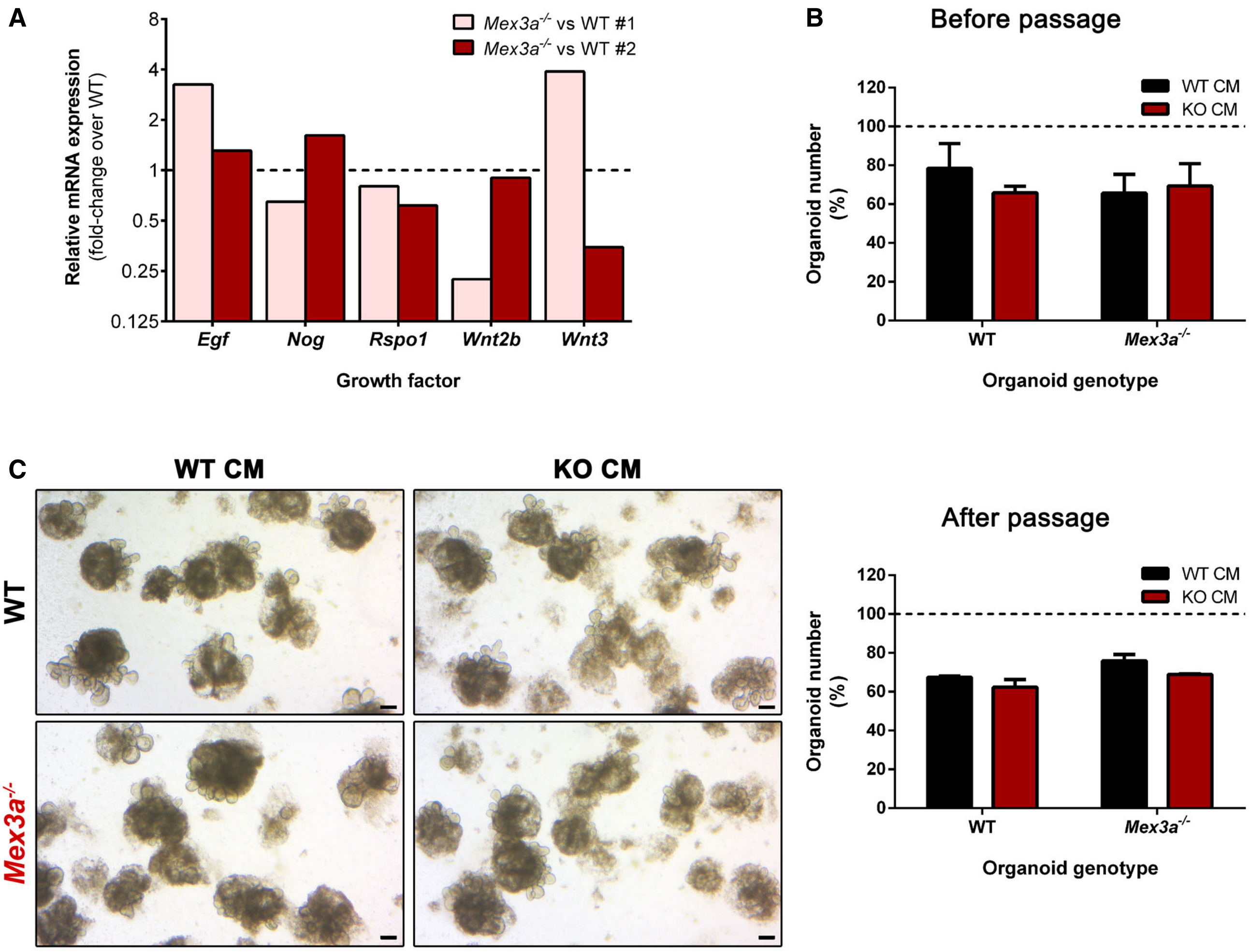

Figure EV4. ***Mex3*a deletion in the mesenchyme does not appear to affect the intestinal phenotype *in vivo* and in organoid cultures.**

A qPCR analysis of the expression level of genes coding for specific growth factors (*Egf*, *Nog*, *Rspo1*, *Wnt2b* and *Wnt3*) in freshly isolated intestinal mesenchymal tissue. Data are presented as the mean fold-change of target gene expression in 2 biological replicates (*Mex3a*$^{-/-}$ vs. WT #1 and *Mex3a*$^{-/-}$ vs. WT #2) of *Mex3a* KO mesenchymal tissue relative to WT (dashed line).

B Number of *Mex3a* null and WT organoids obtained after 6 days in culture with 25% final volume WT or KO mesenchymal cell-derived conditioned media (CM) plus 1% RSPO1-containing ENR medium. Data are presented as the mean percentage plus standard error (n = 2 for each genotype, > 100 organoids counted in total) relative to WT or KO organoids grown in 1% RSPO1-containing ENR only (100%, dashed line).

C Representative phase-contrast microscopy images of *Mex3a* KO and WT organoids obtained 6 days after passaging organoids previously maintained in WT or KO CM. Scale bar, 100 µm. The number of *Mex3a* null and WT organoids obtained in the described conditions is also shown. Data are presented as the mean percentage plus standard error (n = 2 for each genotype, > 100 organoids counted in total) relative to WT or KO organoids grown in 1% RSPO1-containing ENR only (100%, dashed line).

© 2020 The Authors

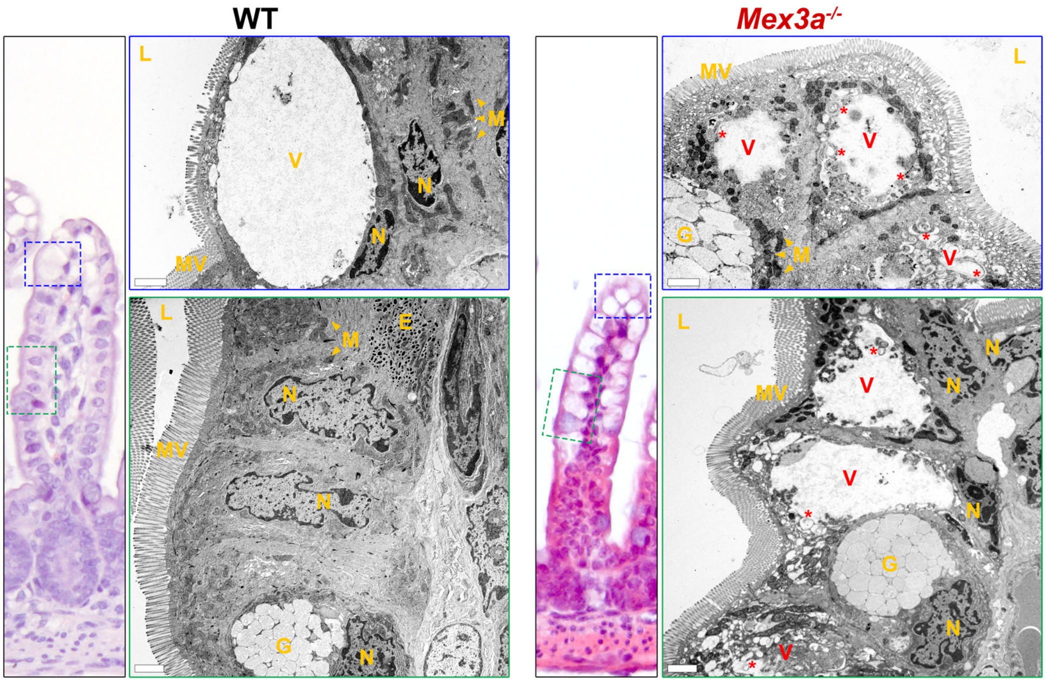

Figure EV5. Transmission electron microscopy (TEM) analysis points to a delayed epithelial turnover in *Mex3a* knockout mice intestine.

TEM analysis shows the presence of suckling-type enterocytes with atypical vacuoles (V) and abnormal ultrastructural features (*) in the *Mex3a* mutant mice ileum. These are observed not only at the tip of the villus (top right panel), but also at the lateral side (bottom right panel). Enteroendocrine cell secretory granules (E); goblet cells (G); lumen (L); microvilli (MV); mitochondria (M); nuclei (N). Scale bars, 2 µm.

 © 2020 The Authors
